# Phase 1 Clinical Trial of *Trametes versicolor* in Women with Breast Cancer

**DOI:** 10.5402/2012/251632

**Published:** 2012-05-30

**Authors:** Carolyn J. Torkelson, Erin Sweet, Mark R. Martzen, Masa Sasagawa, Cynthia A. Wenner, Juliette Gay, Amy Putiri, Leanna J. Standish

**Affiliations:** ^1^Department of Family Medicine and Community Health, Medical School, University of Minnesota, 420 Delaware Street SE, MMC 381, Mayo Building B529, Minneapolis, MN 55455, USA; ^2^Bastyr University Research Institute, Bastyr University, 14500 Juanita Drive N.E., Kenmore, WA 98028, USA

## Abstract

*Introduction*. Orally administered preparations from the *Trametes versicolor (Tv)* mushroom have been hypothesized to improve immune response in women with breast cancer after standard chemotherapy and radiotherapy. *Methods*. A phase I, two-center, dose escalation study was done to determine the maximum tolerated dose of a *Tv* preparation when taken daily in divided doses for 6 weeks after recent completion of radiotherapy. Eleven participants were recruited and nine women completed the study. Each cohort was comprised of three participants given one of three doses of *Tv* (3, 6, or 9 grams). Immune data was collected pre- and postradiation, at 3 on-treatment time points and after a 3-week washout. *Results*. Nine adverse events were reported (7 mild, 1 moderate, and 1 severe), suggesting that *Tv* was well tolerated. Immunological results indicated trends in (1) increased lymphocyte counts at 6 and 9 grams/day; (2) increased natural killer cell functional activity at 6 grams/day; (3) dose-related increases in CD8^+^ T cells and CD19^+^ B cells , but not CD4^+^ T cells or CD16^+^56^+^ NK cells. *Conclusion*. These findings show that up to 9 grams/day of a *Tv* preparation is safe and tolerable in women with breast cancer in the postprimary treatment setting. This *Tv* preparation may improve immune status in immunocompromised breast cancer patients following standard primary oncologic treatment.

## 1. Introduction

*Trametes versicolor (Tv), *also known as *Coriolus versicolor *and commonly called Turkey Tail, is a mushroom species from which preparations have been made that have a long history of use in traditional Asian medicine [1, 2]. Two proteoglycan fractions, polysaccharide-K (PSK) and Polysaccharide-peptide (PSP), are similar hot water extracts of *Tv *with reported anticancer activity [3, 4]. In Japan, PSK is prescribed to cancer patients routinely, both during and after radiation and chemotherapy [5–8]. It is also a common practice among many naturopathic physicians (NDs) and integrative oncologists (MDs) in the US to prescribe whole, freeze-dried *Tv *to breast cancer patients [9]. *Tv *immunologic activity is hypothesized to be the main underlying mechanism responsible for its antitumor effects as well as its impact on survival rates [3, 4]. *Trametes versicolor's *mechanism of action involves enhancement of both innate and adaptive immune responses at least in part via Toll-like 2 receptor agonist activity [10]. Preclinical animal studies and preliminary clinical data support the hypothesis that *Tv* derivatives may be beneficial in treatment of both estrogen receptor negative and positive cancers by mitigating immunologic depressive effects of treatment and enhancing disease-free survival via enhancement of immunological surveillance and overcoming tumor antigen tolerance [5, 6].

Understanding how immunologic factors are influenced by treatments in breast cancer suggests promising areas of focus in developing adjunctive therapies to strengthen antitumor immune responses. Recent data demonstrate that certain classes of chemotherapeutic drugs cause immunogenic tumor cell death, which lead to enhancement of antigen cross-presentation and stimulation of the antitumor immune response. Evidence suggests that NK cells play an important role in prevention of both early and metastatic breast cancer [11]. Some breast cancer patients have been reported to lack NK cell activity against K562 target cells [12]. Current data suggest that breast cancer patients who have completed surgery, chemotherapy, and radiotherapy have immunologic deficit [13, 14]. Andersen et al. have reported that stress levels in breast cancer patients significantly predicted lower NK cell lysis as well as decreased proliferative response to peripheral blood lymphocytes [15]. Our previous observation study done confirmed that lymphopenia and low NK cell activity were present throughout the 6 weeks after the completion of radiation therapy [16].

To better understand the potential benefit of *Trametes versicolor *in women with breast cancer, a dose escalation study to evaluate safety and tolerability was needed. Toward this end, a multidisciplinary team from the University of Minnesota and Bastyr University completed a phase 1 dose escalation trial to determine the safety and maximum tolerated dose of *Tv *in women diagnosed with breast cancer who underwent standard treatment for breast cancer and were willing to participate in a nine-week study following radiation therapy.

## 2. Methods

### 2.1. Study Dose Escalation

A standard phase 1 design was used with 3 subjects per dose level until the MTD (maximum tolerated dose) is reached. Dose escalation schedule for each cohort of *Tv *was a 3 gm (the most commonly used dose); 6 gm; 9 gm; 12 gm; 18 gm; 24 gm. This study recruited through the nine grams cohort only (*N* = 9).

### 2.2. Study Participants

Women between the ages of 21 and 75 years, diagnosed with Stage I, II, or III breast cancer and who had undergone surgery and chemotherapy and were ready to start radiation therapy, were enrolled into the study after providing written consent. Eligibility also included willingness to eat a consistent diet throughout the study, and avoid dietary sources of mushrooms and other herbal products with reported immuno modulating effects during radiation therapy and until the completion of the study. Baseline hematological, renal, and hepatic functions within normal limits were required prior to enrolling in the study. The study was conducted at the Cancer Center at the University of Minnesota in Minneapolis, Minnesota and Bastyr University in Ken more, Washington. The study was approved by the IRB committees at the University of Minnesota and Bastyr University. Between January 2008 and June 2010 eleven women with stage I–III breast cancer met the narrow criteria for this trial, consented to participate, and were enrolled sequentially from the lowest dosage cohort. Three participants in each of the 3 gm, 6 gm, and 9 gm cohorts completed the study. Two of the participants withdrew from the study after visit two because of difficulty with transportation to the clinic. Characteristics of the nine women who completed the study included an age range from 38 to 68 years old; six ER negative and three ER positive; three stage I; two stage II and four stage III breast cancers. One of the women who completed the study did not undergo chemotherapy prior to radiation, and an eligibility protocol violation was reported to the IRB.

### 2.3. Outcome Measures

The primary objective of the study was to evaluate the safety and tolerability of *Trametes versicolor *in women with breast cancer in the postradiotherapy setting. The nine participants in the dose escalation study were monitored for adverse events (AEs) using both clinical and laboratory methods and dose limiting toxicity (DLT) criteria defined as any Grade 2 or greater treatment-related toxicity as scored using the NCI's Common Terminology Criteria for Adverse Events V 3.0 (CTCAE). Women were screened weekly for adverse events during product administration either at their clinic visit or by telephone screening between visits. The secondary aim was to gather preliminary data that compared pre- and postradiation therapy baselines, on-treatment and posttreatment immunologic measures including complete blood count with differential, natural killer (NK) cell activity, T regulatory cell assay, T/B/NK cell subset assay, phagocytic index, and cytokine levels. Statistical analysis, including independent sample *t*-tests and one-way ANOVAs with Turkey *post hoc* multiple comparison, were performed using GraphPad Prism, Version 5.04 (GraphPad Software, Inc., La Jolla, CA). In order to increase power and sample size, data from an observational study (*N* = 14) conducted by our centers since 2006 [16] was combined with the current dose escalation study (*N* = 9). Eligibility criteria were identical for both studies (*N* = 23).

### 2.4. Mushroom Drug Product

*Trametes versicolor *freeze-dried mycelial powder was obtained from Paul Stamets at Fungi Perfecti, Inc., Olympia, WA and was encapsulated by Beehive Botanicals (Hayward, WI). Each capsule contained 500 mg of product. FDA IND approval (# 75,405) was obtained in 2007.

### 2.5. Immune Seizures

#### 2.5.1. Complete Blood Count with Differential

The clinical laboratory tests (CBC, chemistry, serum pregnancy tests and urinalysis) were performed at the Department of Laboratory Medicine at University of Washington for participants recruited at the Bastyr University site and at the University of Minnesota Fairview Diagnostic Laboratory for participants recruited at the U of Minnesota site.

#### 2.5.2. Natural Killer Cell Functional Activity

The immune cells subset measured included NK cell activity, T regulatory cell assay, T/B/NK cell subset assay, phagocytic index, and cytokine levels. Immunological assays were conducted at Bastyr University laboratory for subjects recruited from both sites. Blood collected at the University of Minnesota General Clinical Research Center (GCRC) was shipped overnight to Bastyr University. The quality of transported blood from University of Minnesota to Bastyr was assessed by the following criteria. For specimens older than 48 hours when they arrived at the Bastyr laboratory, the lymphocytes were isolated, and cell viability was determined by trypan blue staining. If the viability was greater than 80%, the assay was performed. If the viability was less than 80%, the specimens were rejected. Peripheral blood mononuclear cells (PBMCs) were isolated by Ficoll-Hypaque gradient (density = 1.070 g/mL), washed twice in PBS, and maintained in RPMI 1640, 10% FBS with 2 mM L-Gln and penicillin-streptomycin (1000 U/mL; 1 mg/mL). NK cell activity of these PBMC samples, as measured by the ability to kill K562 tumor target cells, was assessed in triplicate at the effector to target (E : T) ratios of 50 : 1, 25 : 1, and 12.5 : 1 following published methods [17]. Target cells were labeled with 3,3′-dioctadecyloxacarbocyanine perchlorate (DiOC18) and co-cultured with PBMC effector cells for four hours. A control sample with K562 cells only was included to determine spontaneous target cell death. Following incubation, propidium iodide was added to detect dead cells. The percentage of killed target cells was determined by subtracting the percentage spontaneous lyses from the percent specific lysis (PSL) of respective E : T ratio. To represent standardized NK cell activity, lytic units were calculated by a previously published and validated software program using the following parameters: target per well = 10,000; LU per no. cell = 10^7^; curve maximum = 48; percent lyses = 20 [17]. LU_20_ values, defined as the E : T ratio at which 20% of target cell death occurs, were extrapolated from dose-response curves of PSL versus log E : T ratio for each blood sample assayed [18].

#### 2.5.3. Immunophenotyping PBMC Subsets

PBMC suspensions (5 × 10^5^ cells) were put into 3 tubes, washed in PBS, spun down, supernatants as pirated and cell pellets resuspended in 100 *μ*L PBS. PBMCs in tube 1 were stained by CD3-PC5, CD4-PE, and CD8-FITC; tube 2: CD3-PC5, CD16-FITC, and CD56-PE; tube 3: CD14-PC5. Tubes were vortexed briefly, covered and incubated on ice for 15 minutes, after which cells were washed in cold PBS twice, resuspended to 0.5 mL in PBS and percent PBMC subsets determined by flow cytometric analysis using a Beckman Coulter FC500 and CXP software.

### 2.6. Clinical Trial Protocol

**Figure 1:**
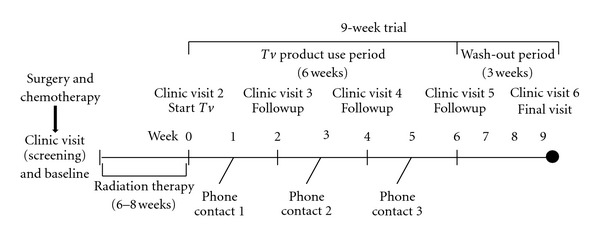
Phase I dose escalation study protocol.

The study duration was nine weeks and started after completion of radiation therapy. It included six weeks of product use followed by a three-week wash outperiod of no product use (Figure 1). Patients were screened and determined eligible for the study after completion of chemotherapy and prior to the initiation of radiation therapy. The study required six visits to the clinical research centers. The first study visit occurred prior to the initiation of radiation therapy, at which baseline labs were drawn. Within the first week following completion of radiation, participants had their second visit at which time labs were drawn, and the nine-week study was initiated. Six weeks of *Tv *product were provided for the participant, who returned to the research center for visit three, four, and five, two weeks apart for lab draw and assessment of product tolerability and adverse events. The final (sixth) visit was at nine weeks, following three weeks off product, for a final lab draw and assessment for any prolonged adverse events.

The dose escalation protocol involved cohorts of a minimum of three participants orally ingesting *Tv *in divided doses daily for six weeks. The first dose cohort took 3 grams/day in two divided doses; the second cohort took 6 grams/day in two divided doses; the third cohort took 9 grams/day in three divided doses. Three grams per day is the most commonly used dose in naturopathic medical practice and as high as 9 grams is used in Japan. The study goal was to determine the maximum tolerated dose (MTD) that demonstrated safety and tolerability (Table 1).

## 3. Results

### 3.1. Adverse Events

Findings from this dose escalation study showed that up to 9 grams/day of *Tv *preparation was well tolerated in women with breast cancer for six weeks in the postradiation treatment setting. There were nine adverse events; seven mild, one moderate, and only one grade 3 adverse events which was an anxiety attack in one participant that was likely unrelated to study medication (Table 2). Other reported mild adverse events included transient heart burn, heart palpitation, constipation, chest pain, fever, radiation dermatitis, and cold/flu symptoms. All three doses were well tolerated, and participants did not experience difficulty in swallowing up to 9 capsules three times daily. Of note is that neither nausea nor GI upset was reported; two side effects were reported in previous clinical trials of other *Tv *extracts.

### 3.2. Immune Response

**Figure 2:**
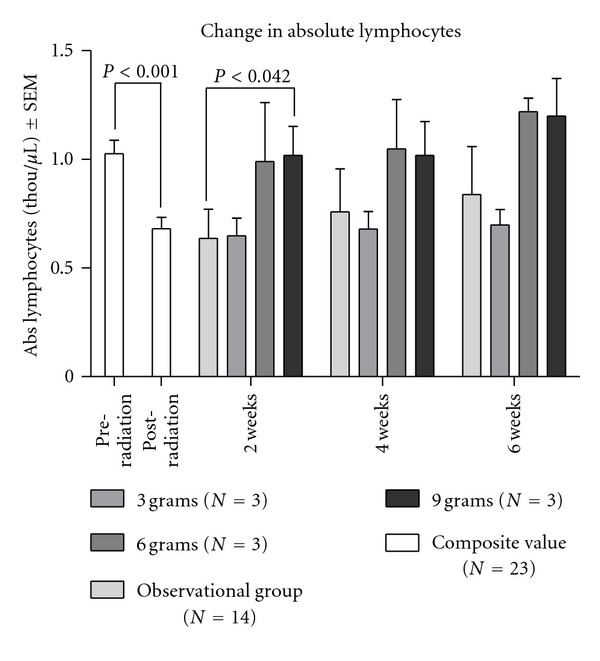


The red blood cell compartment was unaffected by both Radiation Therapy (RT) and *Tv *administration. Absolute red blood cell count, hemoglobin, and hematocrit were normal at visit one, which was the day before RT began, and continued to fall within normal limits throughout the rest of the study. Absolute white blood cell count and neutrophil counts were generally within normal limits for all women both before and after RT and during the weeks after RT while they were taking *Tv *(data not shown). However, absolute lymphocyte counts revealed a different pattern from the other CBC subsets. As eligibility criteria were identical in studies conducted by our centers since 2006, in order to increase power, we combined the data from our observational study (*N* = 14) that showed that RT produced immune defects in women with stage I–III breast cancer [16] with our current dose escalation study (*N* = 9). For the combined number of 23 women in both studies with stage I–III breast cancer status after breast surgery and chemotherapy who then received radiotherapy, lymphocytes were within normal limits before RT but dropped to abnormally low levels after RT. The mean lymphocyte count for all 23 subjects before RT was 1.027 ± 0.298 and dropped 20% after RT to 0.681 ± 0.254, a difference which was statistically significant [*t*(44) = 4.236, *P* < 0.001]. In the dose escalation study, the two higher oral doses (6 and 9 g) of *Tv *resulted in an earlier recovery of lymphocyte counts (Figure 2). The number of lymphocytes gradually recovered for the observational group, but the mean lymphocyte counts remained below those for the 6 and 9 gm *Tv *cohorts at 6 weeks following RT. Due to the small number for each dose cohort (*N* = 3), statistical significance was found only between the observational and 9 g groups at the 2-week postradiotherapy time point. Radiation therapy statistically significantly depressed the absolute number of lymphocytes in women with stage I–III breast cancer who have completed surgery and chemotherapy (see Figure 2).

**Figure 3:**
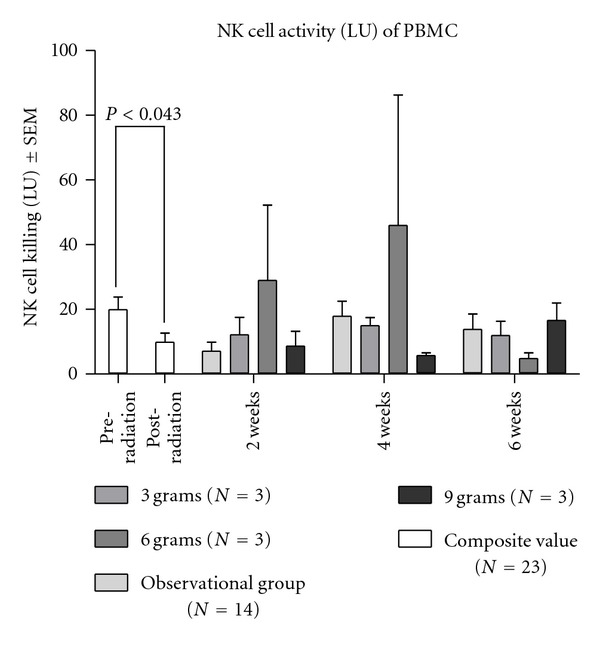


Natural killer cell functional activity was dramatically reduced from pre- to post-RT for all 23 breast cancer patients who completed the first two visits of either the observational study (*N* = 14) or the dose escalation study (*N* = 9).  Figure 3 shows that mean NK cell activity was 19.941 ± 18.959 before RT and decreased to 9.872 ± 13.454 after RT (*N* = 23). This decline was statistically significant (*t*(44) = 2.077, *P* = 0.043). A trend toward increased NK cell activity was observed in the 6 g *Tv *dose cohort.

Radiation therapy statistically significantly depressed the NK cell function represented by the LU, which is calculated by 10^7^ divided doses by the LU_20_. The LU_20_ is the number of effector cells (NK) required to achieve 20% specific lyses on the target cells (K562). Therefore, the smaller the LU_20_, the more effective the NK cells. The interpretation of this result might have been difficult due to differential responses of individuals upon mushroom supplementation. The trend of temporary increase in NK cell activity was observed in the 6 g dosage group (see Figure 3).

**Figure 4:**
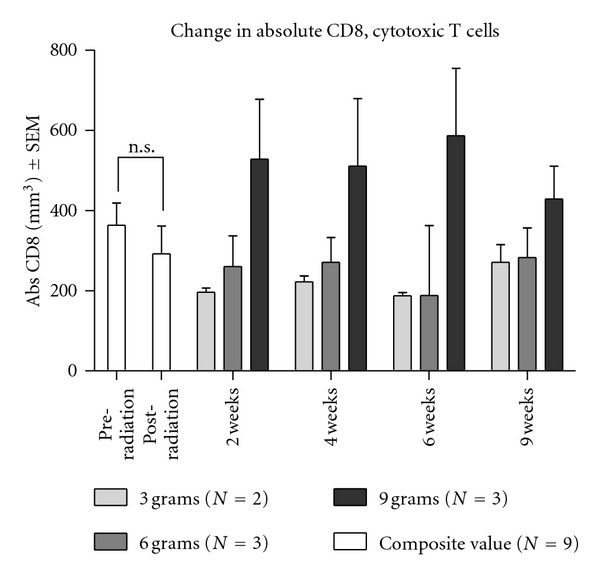


We also measured the effects of radiotherapy and *Tv *administration on T, B, and NK cell populations in the nine patients who completed the phase I dose escalation study. From the WBC and flow cytometry immunophenotyping data, the absolute value (mm^3^) was calculated. Radiotherapy had an insignificant effect on CD4^+^ and CD8^+^ T cell, CD19^+^ B cell, and CD16^+^/56^+^ NK cell populations. Because immunophenotyping was not performed in the observational study, the numbers of composite (pre- and postradiation) scores were of those who participated in the dose escalation study (*N* = 9). The number of CD8^+^ T cells was not statistically different before and after the radiotherapy (Figure 4). However, increases in CD8^+^ T cells and CD19^+^ B cells were observed in peripheral blood for *Tv* supplementation groups. The CD8^+^ T cell counts over the 9-week dose escalation study were enhanced in the 9 gm *Tv *dose cohort compared to both the 3 g or 6 g group. One-way ANOVA was used to analyze the overall difference between dosage groups over the treatment period (2–4–6 weeks). It showed the statistically significant increase in the CD8^+^ cytotoxic T cells for the 9 g group compared to both the 3 g and 6 g group (*F*(2, 6) = 42.04, *P* = 0.0003). The difference between 3 g and 6 g groups was not significant (see Figure 4).

**Figure 5:**
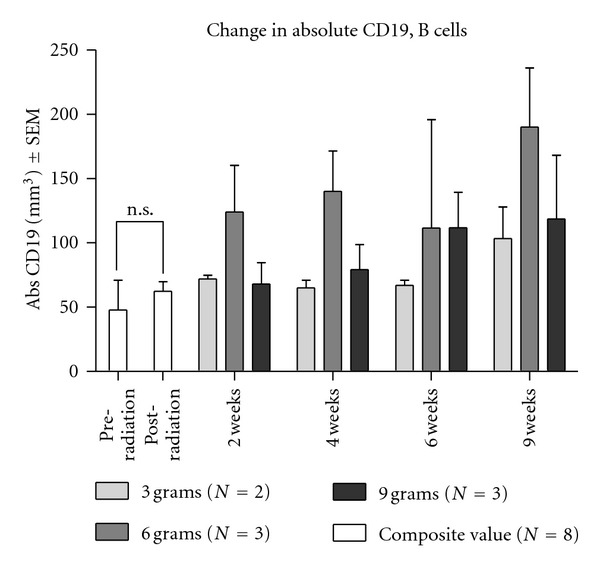


According to the composite scores of eight dose escalation participants, radiotherapy did not significantly alter the B cell count (Figure 5). However *Tv *administration was associated with an increase in CD19^+^ B cells. One-way ANOVA was used to analyze the overall difference between dosage groups over the treatment period (2–4–6 weeks). It showed the statistically significant increase in CD19^+^ B cells for the 6 g dose group compared to the 3 g group [*F*(2, 6) = 6.312, *P* = 0.0334]. The difference between the 3 g and 9 g groups and also the 6 g and 9 g groups was not significant (see Figure 5).

**Figure 6:**
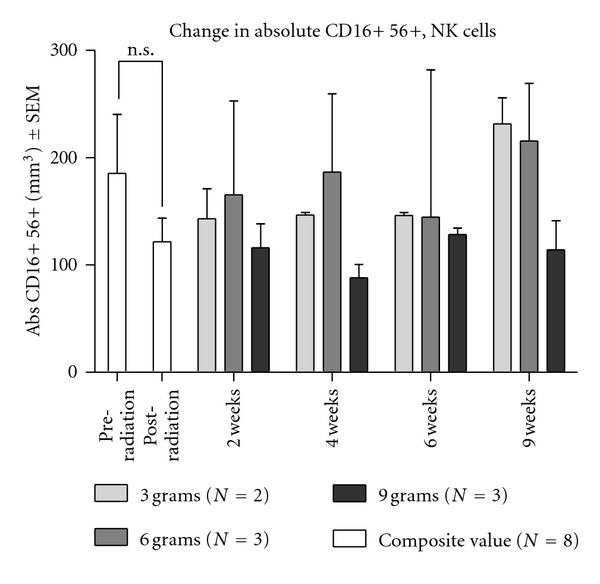


With respect to CD16^+^56^+^ NK cell counts, no significant changes due to either radiotherapy or *Tv *administration were observed (Figure 6). Since NK cell activity was both decreased by RT and increased in the 6 gram *Tv *dose cohort (Figure 3), but NK cell populations appeared not to be significantly influenced by these treatments, the data suggest that enhancement of NK cell activity is not due to treatment-induced alterations in the number of NK cells. NK cell population was unaffected by either radiotherapy or by oral doses of *Tv *(see Figure 6).

## 4. Discussion

Findings from this study establish the safety of oral administration of a *Trametes versicolor (Tv) *preparation at 3, 6, and 9 gram doses, with no serious adverse events of this therapy in women following radiation therapy for breast cancer treatment. The current study confirms our earlier report that standard chest radiotherapy for breast cancer does not affect the red blood cell compartment or neutrophils but does induce specific immune deficiencies including lymphopenia and depressed NK cell functional activity [16, 19]. NK cell therapies are emerging worldwide as promising anticancer treatments, exploiting the fast cytolytic action of NK effectors and their potentially broad applicability against a wide range of malignancies [20–22]. Our new finding is that the loss of NK cell activity appears to be independent of the number of NK cells. Here, we show that RT reduces NK cell activity per NK cell. Higher oral doses of *Tv *at 6 and 9 grams/day were associated with faster recovery of lymphocytes and NK cell activity, as well as increased numbers of CD8^+^T cells and CD19^+^ B cells. There was no evident effect of *Tv *on the number of CD 16^+^/56^+^ NK cells, only on their functional activity. While there is a trend toward higher *Tv *doses having more pronounced immunological activity, this phase I dose escalation trial was not designed to evaluate dose-dependent changes in immune markers. Preliminary data with these small samples of 3 breast cancer patients per dose cohort group showing dose-related trends lead to the testable hypothesis that 6 grams of *Tv *may lead to faster immune recovery after radiotherapy. The results of this phase I trial justify and stand as the platform upon which phase II randomized controlled dose-response trials may proceed.

Although this study showed safety of *Trametes versicolor *to a 9-gram daily dose, the dose escalation study was designed to assess the maximum dose tolerated (MDT); therefore this study did not determine safety and tolerability at the highest dose. Recruitment of women into a 12-gram cohort and higher was not achieved because of multiple limiting factors. First, women had to meet very strict eligibility criteria to enroll in the study and be undergoing surgery, chemotherapy, and radiation in their treatment protocol. Currently, many women with a breast cancer diagnosis do not receive a triple therapy regimen. Many women are diagnosed with stage 1 breast cancer and undergo lumpectomy and radiation, and if their oncotype score is low, they do not receive chemotherapy. Additionally, many women were recruited into other research studies and were saturated with requests to enroll in additional studies. Most importantly, we found that women after completing a lengthy treatment regimen of chemotherapy and radiation were reluctant to participate in a nine-week study that required six additional visits to the University.

The phase I data suggest that *Tv *is a safe immunotherapy for breast cancer patients that may correct radiotherapy-related immune defects. Based on our findings, *Tv *mushroom therapy orally administered in the postradiotherapy setting may enhance lymphocyte numbers and NK cell tumoricidal activity. Relapse after primary breast cancer treatment may be related to defects in the innate and adaptive immune system. Research by our center continues to indicate that *Trametes versicolor *represents a novel immune therapy with significant applications in cancer treatment.

## Figures and Tables

**Table 1:** Phase I dose escalation.

Cohort	*Tv *g/day	Dose escalation timing (using escalation decision rule in next box)
1	3	Starting dose (dose used in clinical practice)
2	6	Begin after a minimum of 3 subjects in group 1 who have completed 2 weeks of product with no dose limiting toxicity (DLT—defined as any treatment related toxicity > grade 1)
3	9	Begin after a minimum of 3 subjects in group 2 who have completed 2 weeks of product with no DLT

**Table 2:** Adverse event reporting.

Grade	Frequency	Dose	Description	Relation	Expectation
Mild	7	3 g	Heartburn	Possibly related	Unexpected
		3 g	Heart palpitations	Unlikely related	Unexpected
		6 g	Constipation	Unlikely related	Unexpected
		6 g	Chest pain	Possibly related	Unexpected
		6 g	Fever with concomitant R dermatitis (2)	Unlikely related	Unexpected
		9 g	Cold or Flu-like symptoms	Unlikely related	Unexpected

Moderate	1	6 g	Fatigue; secondary to UT infection	Unlikely related	Unexpected

Severe	1	6 g	Anxiety event	Possibly related	Unexpected

Total	9
